# Arp2/3 Branched Actin Network Mediates Filopodia-Like Bundles Formation *In Vitro*

**DOI:** 10.1371/journal.pone.0003297

**Published:** 2008-09-29

**Authors:** Yaron Ideses, Yifat Brill-Karniely, Lior Haviv, Avinoam Ben-Shaul, Anne Bernheim-Groswasser

**Affiliations:** 1 Ben-Gurion University of the Negev, Beer-Sheva, Israel; 2 The Hebrew University, Jerusalem, Israel; Geroge Mason University, United States of America

## Abstract

During cellular migration, regulated actin assembly takes place at the cell leading edge, with continuous disassembly deeper in the cell interior. Actin polymerization at the plasma membrane results in the extension of cellular protrusions in the form of lamellipodia and filopodia. To understand how cells regulate the transformation of lamellipodia into filopodia, and to determine the major factors that control their transition, we studied actin self-assembly in the presence of Arp2/3 complex, WASp-VCA and fascin, the major proteins participating in the assembly of lamellipodia and filopodia. We show that in the early stages of actin polymerization fascin is passive while Arp2/3 mediates the formation of dense and highly branched aster-like networks of actin. Once filaments in the periphery of an aster get long enough, fascin becomes active, linking the filaments into bundles which emanate radially from the aster's surface, resulting in the formation of star-like structures. We show that the number of bundles nucleated per star, as well as their thickness and length, is controlled by the initial concentration of Arp2/3 complex ([Arp2/3]). Specifically, we tested several values of [Arp2/3] and found that for given initial concentrations of actin and fascin, the number of bundles per star, as well as their length and thickness are larger when [Arp2/3] is lower. Our experimental findings can be interpreted and explained using a theoretical scheme which combines Kinetic Monte Carlo simulations for aster growth, with a simple mechanistic model for bundles' formation and growth. According to this model, bundles emerge from the aster's (sparsely branched) surface layer. Bundles begin to form when the bending energy associated with bringing two filaments into contact is compensated by the energetic gain resulting from their fascin linking energy. As time evolves the initially thin and short bundles elongate, thus reducing their bending energy and allowing them to further associate and create thicker bundles, until all actin monomers are consumed. This process is essentially irreversible on the time scale of actin polymerization. Two structural parameters, *L*, which is proportional to the length of filament tips at the aster periphery and *b*, the spacing between their origins, dictate the onset of bundling; both depending on [Arp2/3]. Cells may use a similar mechanism to regulate filopodia formation along the cell leading edge. Such a mechanism may allow cells to have control over the localization of filopodia by recruiting specific proteins that regulate filaments length (e.g., Dia2) to specific sites along lamellipodia.

## Introduction

Actin polymerization at the plasma membrane results in the formation of cellular protrusions known as lamellipodia or filopodia, which mediate cell migration [1]–[3]. The distinct organization and generation of filaments in each structure uses a different mechanism to produce mechanical force. In the lamellipodia, the actin filaments organize into a flat 2D branched network [2], [4] whereas in the filopodia they are assembled into long, parallel, closely packed bundles [5]–[7]. Different proteins control the assembly of these structures; in the lamellipodia, the branched nucleation is driven by activation of the Arp2/3 complex [8] by Wiskott-Aldrich syndrome protein family [9], [10] (WASP), followed by filament elongation and barbed-end capping by capping proteins (CP) [11]. Formin and Ena/VASP proteins are concentrated at the tips of filopodia [12]–[15] promoting the continuous elongation of filaments [3], [16] and their successive binding by fascin, which crosslinks actin filaments of the same polarity into the bundles constituting the “body” of filopodia [17]. In both structures, the barbed (i.e., the fast growing) ends of actin filaments point towards the plasma membrane [18].

Most cultured animal cells assemble both lamellipodia and filopodia. Some cells, like dendritic cells, are dominated by filopodia [16] while keratocytes grow exclusively lamellipodia [19]. It is still not fully understood what determines the *in vivo* preference to lamellipodia vs. filopodia, and why certain cells are dominated by only one type of structure. So far, two mechanisms were suggested to explain the formation of filopodia [20]. In the “*de novo* filopodia nucleation” model, actin filament nucleation and elongation is mediated by Dia2 proteins [15], [21]. According to this mechanism bundles do not emanate from lamellipodia and Arp2/3 complex is not essential for filopodia formation. In the second mechanism, known as the “convergent elongation” model, filopodia emerge from the Arp2/3 lamellipodial network [22]–[25]. During formation of filopodia, lamellipodial filaments associate at their barbed ends with mDia2 or VASP, elongate continuously and gradually converge into filopodia bundles, by the cross-link of fascin. In contrast to the first mechanism, here filopodia are anchored to lamellipodia. Both systems can be reconstituted *in vitro*. This work primarily focuses on the “convergent elongation” model.

Notwithstanding recent advances in understanding the dynamic organization of lamellipodia and filopodia protrusions, it is still not fully understood how cells control the transition between these structures, what directs the localization of filopodia formation along the cell leading edge, and how their thickness is regulated. It is expected that the emergence of filopodia from lamellipodia would be strongly affected by the properties (e.g., the density and length of filaments) of the branched lamellipodial network from which they emanate. The concentration of Arp2/3, which strongly affects these structural properties, was recently observed to have a dramatic influence on the formation of filopodia *in vivo* [25]. The abundance of fascin, which is the driving force for bundling is also expected to affect bundles formation. Yet, it is still not clear how the structure of lamellipodia and the fascin concentration control filopodia formation.

To resolve these issues, it is essential to understand the mechanisms underlying the formation of lamellipodia and filopodia and evaluate the factors controlling their structure and dynamics. To this end, in the present work we study a system containing the major proteins participating in the assembly of lamellipodia and filopodia: a) the constitutively active VCA [26] (or WA [8]) domain of WASp; b) Arp2/3 complex; c) fascin, and d) actin monomers. This model system enables us to control the concentrations of the various proteins, and to observe actin organization in bulk. Recently, we have demonstrated the feasibility of using such a simple reconstituted system for studying the formation of lamellipodia and filopodia-like structures *in vitro* [27]. In this early work, the roles of Arp2/3 complex and fascin in the transition from the Arp2/3 branched network to filopodia-like bundles was addressed only briefly. Also, no attempt was made to model this transition nor to resolve the primary factors controlling the bundling process.

Our goal in the present work is to study in detail the self-assembly characteristics of actin in the presence of variable amounts of Arp2/3 complex and fascin. We will show that in the absence of fascin, actin organizes into dense 3D, aster-like, structures composed of branched networks of actin filaments [27]. The growth of the network advances with the barbed ends of the peripheral actin filaments pointing on average outward, as also demonstrated in the simulations [27]. Addition of fascin leads to the formation of star-like structures, where actin/fascin bundles are nucleated and emanate radially from the branched network core. The polarity of the actin filaments during the transition is preserved, as in the transition from lamellipodia to filopodia [27]. Our experiments show that in the early stages of actin polymerization, Arp2/3 mediates the formation of the dense and highly branched aster-like network, whereas fascin is rather passive. Fascin becomes dominant at a later stage of actin polymerization, when the (sparsely branched) filaments in the periphery of the aster become long enough, so that the energy gained due to actin-fascin-actin links between neighboring filaments overcomes the unfavorable bending energy required to bring them into contact. As time evolves the initially thin bundles elongate, thus lowering their bending energy thereby enabling their association into thicker bundles, and so on until no more actin monomers are left.

This picture of stepwise bundle elongation and thickening underlies our structural-energetic model of bundle formation and growth. Using reasonable approximations for the bending energies of actin filaments and bundles, and the cohesive energy due to fasin-actin bonds we can explain the dependence of the thickness and length of the mature bundles upon [Arp2/3]. More explicitly, using Kinetic Monte Carlo (KMC) simulations we can model the nucleation and growth of asters and derive their structure as a function of time thus obtaining two central structural parameters: the length, *L*, of filament tips at the aster periphery, and the spacing, *b*, between their origins (i.e., their pointed ends, anchored at Arp2/3 branch points). These parameters, which determine the initial conditions at the onset of bundling, depend sensitively on [Arp2/3]. As we shall see, our theoretical scheme, which combines the KMC and the stepwise bundle assembly model, accounts adequately for the experimentally observed dependence of bundle structure on the initial concentration of Arp2/3 in the system.

## Results

The first part of this section describes the three types of structures, i.e., asters, stars and network of bundles (a phase of actin-fascin bundles) that were experimentally observed and the transition between them. We also present a phase diagram of the system to illustrate the regions of existence of the three types of structures as a function of the initial [Arp2/3] and [fascin]. In the second part, we focus specifically on the transition from asters to stars, as it mimics the transition from lamellipodia to filopodia in cells. Special attention is given to evaluating the roles of [Arp2/3] and [fascin] on filopodia formation and dimensions. In the last part of this section we describe our theoretical model for bundle assembly and apply it to explain our experimental results.

### Fascin mediates structural transition of actin self-assembly

**Figure 1:**
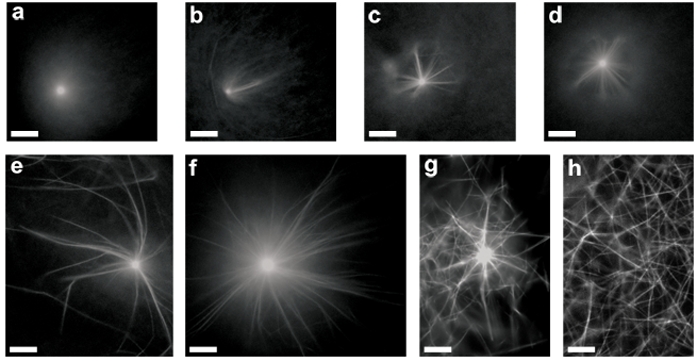
Phase transition as a function of fascin concentration. Conditions are 7 µM G-actin, 6.25 nM Arp2/3, and 12.5 nM GST-VCA. Fascin concentration is: (a) 3 nM, (b) 4 nM, (c) 5 nM, (d) 7 nM, (e) 50 nM, (f) 200 nM, (g) 500 nM, and (h) 3 µM. At very low concentrations of fascin asters are formed. Transition to star-like structures occurs above a fascin concentration of 4 nM (b). The density and the length of the bundles emanating from the star core increase with fascin concentration (b–f). Above a certain fascin concentration of 0.5 µM (g) the size and the number of stars decreases; the stars coexist with an entangled network of actin/fascin bundles, seen in the image background. At 3 µM stars do not form anymore; the system is composed solely of entangled network of actin bundles (h). Bar is 10 µm.

First, we aimed at studying the self–assembly of actin in the presence of increasing amounts of fascin, at a given [Arp2/3]. At low Arp2/3 complex concentration of 6.25 nM (the [VCA]/[Arp2/3] ratio was kept equal to two in all experiments) two structural transitions are observed, the first, from ‘asters’ to ‘stars’, and the second, from ‘stars’ to ‘network of bundles’ (Fig. 1). At a very low fascin concentration (3 nM) the structure of actin network is dominated by Arp2/3 nucleation and branching activity–the system organizes into diffuse highly branched 3D aster-like structures (Fig. 1a), similar to those formed in the absence of fascin [27]. Surprisingly, a minute concentration of fascin ([fascin] = 4 nM) was sufficient to mediate the nucleation and growth of bundles emanating from branched asters' cores. Fig. 1b shows such a star-like structure after reaching final dimensions. The bundles grow with their barbed ends pointing outward, as previously demonstrated [27]. The time span of this process ranges between few to ten minutes, depending on [Arp2/3] and [fascin] (data not shown). In this work all the experimental data analyses were performed for systems that have reached their final state.

Gradual addition of fascin, from 0.005 to 0.2 µM, increases the number of bundles emanating from the asters core as well as their length (Figs. 1c–1f). Above 0.2 µM of fascin, the system is dominated by the bundling activity of fascin. Most of the actin appears to be located in actin/fascin bundles residing in the bulk solution (see image background, Fig. 1g; [fascin = 0.5 µM]). The stars that still exist are composed of fewer and shorter bundles. Eventually, at high enough [fascin] (3 µM) stars no longer form; instead, a network of actin/fascin bundles is generated (Fig. 1h).

### [Arp2/3] controls fascin ability to induce structural changes

**Figure 2:**
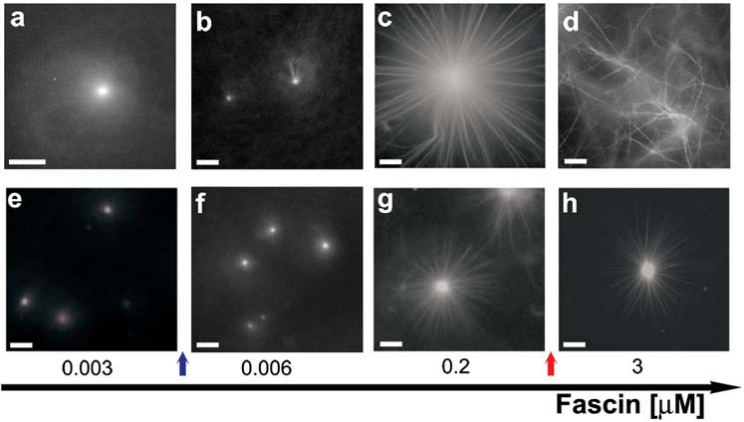
Transition from aster to star-like to network structures induced by fascin depends on Arp2/3 concentration. Conditions are: 7 µM G-actin, upper line (a–d) 12.5 nM Arp2/3 complex, and 25 nM GST-VCA and bottom line (e–h) 100 nM Arp2/3, and 200 nM GST-VCA. Transition from aster to stars and from stars to network: occurs between 3 to 6 nM and between 0.2 to 3 µM at 12.5 nM [Arp2/3] (blue and red arrows, respectively). At 100 nM [Arp2/3] only the transition from aster to star is visible; transition to a network structure is not visible in these fascin concentration ranges. Bars are 10 µm (a–h).

Structural transformations mediated by fascin seem to be facilitated when the initial [Arp2/3] was reduced. The top and bottom panels in Fig. 2 distinguish between fluorescence microscopy images corresponding to mixtures containing low ([Arp2/3] = 12.5 nM) and high ([Arp2/3] = 100 nM) initial Arp2/3 contents, respectively. In both cases [fascin] varies in the same manner. Initially, at a very low [fascin] of 3 nM (Figs. 2a and 2e), actin organization is fully dominated by Arp2/3 complex activity; diffuse asters are formed, whose number increases with the initial [Arp2/3]. These asters are smaller and their branched actin cores are denser (Fig. 2e) compared to those formed at the lower [Arp2/3] (Fig. 2a). Increasing the concentration of fascin to 6 nM (blue arrow) induced a transition from asters to stars when [Arp2/3] = 12.5 nM (Fig. 2b; Figs. S1a and S1b show that the transition occurs between 5 nM and 6 nM of fascin). On the other hand, 6 nM fascin is not sufficient to induce bundle formation in the [Arp2/3] = 100 nM system (Fig. 2f). In this latter case, more fascin (7 nM) is required to nucleate bundles from the branched network core (see also Figs. S1c and S1d).

Further addition of fascin induces the formation of additional and longer bundles, resulting in the appearance of fully developed stars, as shown in Figs. 2c and 2g. The total number of bundles nucleated per star in the low [Arp2/3] (12.5 nM) case appears to be larger than in the high [Arp2/3] (100 nM) case. Moreover, the bundles in the low [Arp2/3] system look both thicker (i.e., they comprise a larger number of actin filaments) and longer. Clearly then, the initial concentration of Arp2/3 in solution plays a major role in determining the conditions for the fascin-mediated assembly of actin bundles, thus (albeit indirectly) controlling bundles thickness and length.

Finally, when the amount of fascin was further elevated to 3 µM (red arrow), a transition from ‘stars’ to ‘a network of bundles’ was observed in the 12.5 nM Arp2/3 system (Fig. 2d). In contrast, the system with 100 nM Arp2/3 remained unchanged (Fig. 2h) and did not show any structural transition up to 6 µM fascin (data not shown). Actually ‘networks of bundles’ did not appear in any of the systems containing more than 25 nM of Arp2/3 complex, regardless of [fascin].

**Figure 3:**
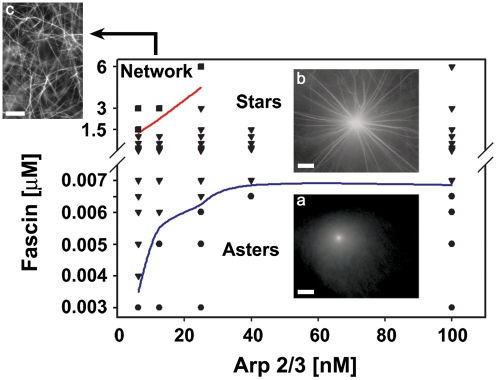
Phase diagram for actin-fascin-arp2/3 complex. Condition is: 7 µM G-actin, Arp2/3 complex and fascin concentration was changed according to the graph. Condition for images is: 3 nM, 200 nM and 3 µM fascin; 6.25 nM, 12.5 nM and 6.25 nM Arp2/3 complex (a, b and c, respectively). Experimental points demonstrate different type of structures formed; these are represented by squares - entangled network, triangles - stars and circles - asters. Phase changes from asters to stars (blue line) and from stars to entangled bundle network (red line). Lines of phase separation are plotted as averages of two points of different structures. For low concentration of Arp2/3 complex phase transition is more abrupt for the same change in fascin concentration. No transition to entangled network was visible for high concentrations of Arp2/3 complex.

In Fig. 3 we present a phase diagram of the system, depicting the regions of existence of the three types of experimentally observed structures, as a function of the initial [Arp2/3] and [fascin]. The data shown here corresponds to experiments conducted at [G-actin] = 7 µM; a similar qualitative behavior was observed for the other concentrations of actin we have tested, ranging between 1 µM and 5 µM (data not shown). Careful inspection shows that below 3 nM of fascin only aster-like structures form (circles), regardless of [Arp2/3]. Increasing [fascin], for a given amount of Arp2/3 complex, induces structural transitions into stars (inverted triangles). The amount of fascin required to mediate this transition increases nonlinearly with [Arp2/3], saturating above 40 nM Arp2/3, (the blue curve marks the approximate boundary between the two regions). While only few nano-molars of fascin are sufficient to induce the transition from asters to stars, the subsequent transition to ‘networks’ (squares) requires much larger amounts of fascin (several µM; the red curve marks the approximate boundary between the two regions). We note that a transition to ‘networks’ is observed only for sufficiently low [Arp2/3] (<25 nM), within this region, the amount of fascin required for the nucleation of bundles increases monotonically with Arp2/3 content.

### Aster to star transition–Experimental results

#### The densities of asters and stars depend on [Arp2/3] but not on [fascin]

**Figure 4:**
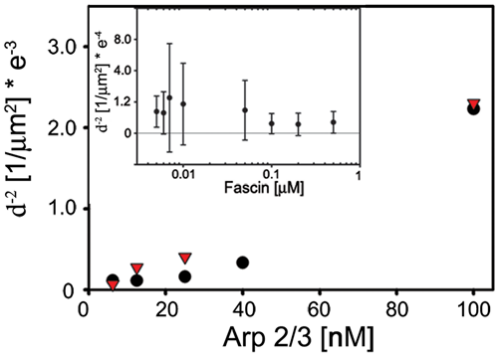
The surface density of objects (asters or stars) is controlled solely by Arp2/3 and not by fascin. Conditions are: 7 µM G-actin; fascin concentration of 5 nM (triangles) and 200 nM (circles); and variable amount of Arp2/3 complex: 6.25, 12.5, 25, 40, and 100 nM. The [VCA]/[Arp2/3 complex] = 2 in all experiments. We observe a monotonic growth in density of objects with Arp2/3 complex concentration. The density of objects doesn't show a dependence on fascin concentration (inset, a half log plot is given in order to see clearly all experimental data points). Error bars represent standard deviation from average values.

To test our assumption that each star originates from a preformed aster, we have measured the number of “aggregates” (i.e., asters or stars) per unit area (surface density), within the 1 µm section of the bulk sample observed. The surface density of aggregates *σ* = *d* ^−2^ was evaluated by measuring the average distance, *d*, between adjacent aggregates. Keeping a constant ratio [VCA]/[Arp2/3] = 2, we have measured *d* ^−2^ for several values of the initial [Arp2/3] and [fascin] (Fig. 4). For clarity we present only two concentrations of fascin: 5 nM (triangles) and 0.2 µM (circles). Data points taken at 5 nM fascin correspond to density of asters, while those taken at 0.2 µM fascin are associated with density of stars. In both cases, *d* ^−2^ increases monotonically with [Arp2/3]. In contrast, [fascin] does not appear to affect *d* ^−2^ (see inset; conditions: [Arp2/3] = 12.5 nM), indicating that the density of aggregates is dictated primarily by the amount of Arp2/3 complex in the system. These results support our previous findings that Arp2/3 complexes are aster nucleators and that stars develop from preformed asters [27]. This is consistent with our present conclusion that, at early times, actin polymerization is governed by Arp2/3 nucleation and branching. Fascin comes into play and becomes dominant in star formation only later, when the structural properties of the aster enable the onset of filament bundling.

#### The length of bundles in stars depends on Arp2/3 and fascin concentrations

**Figure 5:**
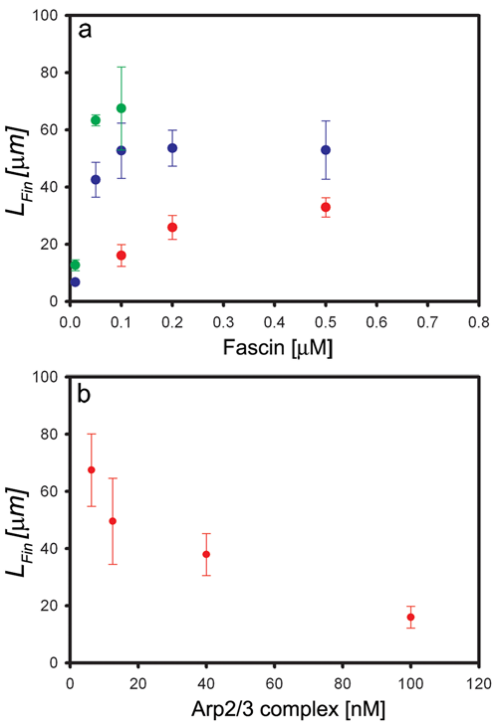
Mean star-like bundles' length, *L_Fin_*, dependence on the [Arp2/3] and [fascin]. (a) Conditions are: [G-actin] = 7 µM; [Arp2/3] = 6.25 nM (green dots), 25 nM (blue dots), and 100 nM (red dots). For all three cases, the mean bundles' length, *L_Fin_*, increases with [fascin]. (b) Conditions are: 7 µM G-actin; 100 nM fascin and variable amounts of Arp2/3 complex. The data shows that the average of bundles length, *L_Fin_*, decrease with [Arp2/3] monotonically (error bars are ±SD).

For all the concentrations of Arp2/3 that we have tested, the mean final length of bundles, *L_Fin_*, was found to increase with [fascin], reaching asymptotically a maximal ([Arp2/3] dependent) value, 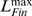 (Fig. 5a). The system appears more sensitive to fascin addition when [Arp2/3] is lower (as reflected by the steeper slopes in Fig. 5a). Consequently, the amount of fascin required to reach 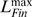 decreases with the decrease in [Arp2/3]. In contrast, for a fixed concentration of fascin, we found that *L_Fin_* decreases monotonously with [Arp2/3], (as seen for [fascin] = 100 nM in Fig. 5b). The inverse correlation between *L_Fin_* and [Arp2/3] results from the reduced amount of actin monomers available for bundle growth when [Arp2/3] is increased, as discussed in more detail in the theory section below.

### Theoretical model and analysis

#### Bundle formation: A simple model and Kinetic Monte Carlo Simulations

Our experiments show that upon mixing actin monomers with Arp2/3 complex, VCA and fascin molecules, the first structures to appear in solution are dense and highly branched aster-like networks of actin filaments [27]. The asters grow radially outwards, and since Arp2/3 joins both old and newly formed filaments, the density of branching points and hence also of polymerized actin is highest in the center of the aster, decreasing gradually towards its periphery, where the average spacing between adjacent branches is relatively large [27]. To associate into bundles, nearby filaments are generally required to bend towards each other. Inside the aster core, owing primarily to excluded volume interactions between the rigid, dense and highly branched network, filament bending involves a prohibitively large energetic penalty and bundle nucleation is thus highly unlikely.

Bending the filaments emanating from the surface of the aster is easier, yet two conditions must be fulfilled to enable the onset of bundle formation. First, *L*, the average length of the filament tips in the aster periphery, should be larger than the distance, *b*, between their pointed ends (which mark their origins). The second requirement is that the energy gained due to fascin links between neighboring filaments should exceed the bending energy associated with bringing them into contact. Both conditions are met at some time *t_i_*, in the late stages of aster growth, owing mainly to the decrease in [Arp2/3] and hence the increase in *L*. Qualitatively, this explains why in the early stages of actin polymerization the (dense aster-like) structure of the actin network is dominated by Arp2/3, with fascin playing a rather passive role. However, once the peripheral filaments are long enough, fascin comes into play–linking the filaments into bundles, whereas branching essentially ceases; especially along and within the bundles, because the (already largely depleted) Arp2/3 complexes cannot penetrate the dense bundle.

Our goal in this section is to explain the experimental observation that bundles are thicker and longer when the initial [Arp2/3] is low (see Figs. 2 and 5). Qualitatively, this behavior is understood based on the fact that since Arp2/3 is an aster nucleator, so that for a given concentration of G-actin, higher [Arp2/3] results in the appearance of more asters in solution. Thus, on average, fewer actin monomers participate in the growth of each aster. Also, since the asters formed are denser, fewer monomers are later available for bundle growth, resulting in relatively short bundles, as observed in our experiments (Figs. 2 and 5). It should be noted that in living cells, the plasma membrane also affects the length of filopodia, as was previously analyzed theoretically [23], [28]. Furthermore, in our system, owing to the large energy penalty associated with the bending of short bundles towards each other, and because the bending energy increases rapidly with bundle thickness (see below), when [Arp2/3] is high, association of bundles is energetically costly, and the mature bundles are both shorter and thinner than in the case of low [Arp2/3].

**Figure 6:**
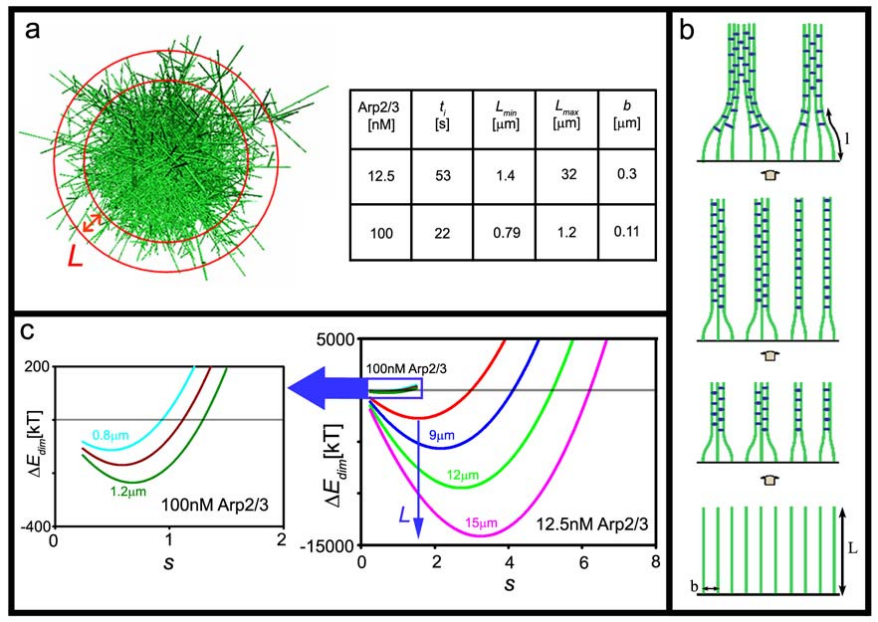
Analytic Model and KMC simulations emphasize the role of Arp2/3 in determining the thickness of bundles. (a). Left: snapshot of an aster taken from the simulations. Filaments originating in the periphery shell of the aster might bend towards each other and link into bundles. Right: Values of *L_min_*, *L_max_* and *b* that were taken from the simulations at *t_i_*, for the two [Arp2/3] tested. (b). Bundles formation from the branched network occurs by the elongation–association model. The length *l* represents the bent portion of a filament/bundle. (c) The change in energy caused by bending and linking of two adjacent bundles, as a function of their number of shells s (which also corresponds to the bundles' thickness), for several values of *L*. For [Arp2/3] = 12.5 nM: *L* = 6 µm (red), 9 µm (blue), 12 µm (light green), and 15 µm (pink), and *b* = 0.3 µm. For [Arp2/3] = 100 nM (magnified in the black box): *L* = 0.8 µm (light blue), 1.0 µm (brown), and 1.2 µm (green), and *b* = 0.11 µm. In both cases [G-actin] = 7.5 µM.

The structural properties of the asters at the onset of bundle formation, primarily the distances *b* and *L* defined above, enter our model as input parameters. Numerical estimates of these quantities were derived using our 3D KMC simulations of aster growth. To derive the aster structural characteristics relevant to the experiments reported here, we have used a limited version of the simulation, which includes only actin polymerization and Arp2/3 mediated branching [27], (see also Appendix S1, in “Supporting information”). A simulation snapshot of an aster is shown in Fig. 6a. It should be noted that (at least presently) these simulations do not account for filament bending, and thus cannot describe bundle formation; they are used here only to provide the initial *L* and *b*.

At the onset of the aster-to-star transition the radius of the aster is generally much larger than both *b* and *L*. Since bundles are formed by the association of neighboring filaments whose origins are just a few *b*'s apart, we ignore the curvature of the aster's surface and treat it as being planar. It may be noted, however, that once bundles become much larger than the aster core, surface curvature correction should (and can) be taken into account.

Assuming that all the *M* filaments participating initially in bundle formation are of the same length *L*, and organize into *M/N* identical bundles, each of which consists of *N* filaments, the total energy of the system is given by *E_tot_* = (*M*/*N*)*E_bundle_*(*N*;*L*,*b*). The bundle energy *E_bundle_*(*N*;*L*,*b*) includes the cohesive energy due to the fascin-mediated linking of filaments, the bending energy cost of bringing the filaments into contact, and the (unfavorable) surface energy of a finite size bundle. All these contributions depend on *N*, and parametrically on *L* and *b* (Additional contributions, such as the entropy loss experienced by the filaments upon bundling can be neglected). If bundle elongation were slow compared to filament (and bundle) binding-unbinding events, then the optimal bundle size *N* ^*^ could be derived by minimizing *E_tot_* with respect to *N*. Using reasonable models for the bending, cohesion and surface energies one can show that the resulting *N* ^*^ is indeed larger when [Arp2/3] is lower. It must be noted, however, that in this scheme the optimal size and length of bundles is determined by the principles of equilibrium thermodynamics. However, kinetic estimates of the rates of bundle dissociation (based on calculations of filaments dimerization time, [29], [30] (data not shown) indicate that once bundles are formed, their life time is significantly longer than the time scale of actin polymerization, so that filament and bundle associations are practically irreversible on the time scale of our present experiments.

Based on this notion we propose here an alternative, stepwise, mechanism whereby single filaments first associate into thin bundles (Fig. 6b). The nascent thin bundles are hard to bend, but after elongating their bending and hence fascin-mediated association into thicker bundles becomes easier. This scenario may continue, leading to stepwise bundle thickening. It should be noted that *in vivo* observations of bundles roots also indicate that bundles initiate in a gradual process of elongation and thickening [5]. In our *in vitro* experiments, this process terminates when there are essentially no more actin monomers in the system.

Suppose for simplicity that all possible bundles have a circular cross section consisting of a central straight filament surrounded by *s* concentric shells of filaments; *s* = *R*/*d* where *R* is the radius of the bundle, and *d* is the average distance between adjacent filaments. Fig. 6c shows Δ*E*_dim_(*s*), the energy change corresponding to the dimerization of two bundles of the same size *s* containing *N*≈*πs* ^2^ filaments, into a thicker bundle comprising 2*N* filaments. This simplified calculation ignores many association processes other than bundle doubling (e.g., monomer-dimer, monomer-tetramer binding, etc.) Yet, it captures, at least qualitatively, the kinetic-energetic picture of bundle growth.

Results are shown for two of the initial [Arp2/3] studied experimentally (12.5 and 100 nM), and several representative values of *L* which, according to our aster growth simulations, represent relevant filament lengths for the time and concentration scales of the experiments. More specifically, the range of *L* considered is *L_min_*([*Arp2*/*3*])<*L*<*L_max_*([*Arp2*/*3*]), where *L_min_*([*Arp2*/*3*]) is the length of filaments at the time *t_i_*, marking the onset of bundling, and *L_max_*([*Arp2*/*3*]) is the length of bundles when all G-actin was consumed. Our simulations show that there are about 10^6^ free monomers per aster at *t_i_* (see methods) when [Arp2/3] = 100 nM, compared to ∼10^8^ monomers for 12.5 nM. In both cases, the number of filaments in the aster periphery is about 5000 at *t_i_*. Thus *L* ranges between 0.8–1.2 µm for 100 nM Arp2/3. In the 12.5 nM Arp2/3 *L* is much larger: 1.5–30 µm.

Association of two adjacent bundles of thickness *s* to form a thicker one is energetically favorable when Δ*E*_dim_(*s*) is negative. Fig. 6c shows, for instance, that for [Arp2/3] = 12.5 nM, bundles of length 9 µm that are composed of 5 shells of filaments will not associate with each other. However, once their length increases to, say, *L* = 12 µm, they tend to stick to each other since Δ*E*_dim_ becomes negative. From Fig. 6c we note that for both values of [Arp2/3] considered, as *L* increases thicker bundles become energetically favorable; the optimal bundle thickness (corresponding to the minimum of Δ*E*_dim_(*s*)) increases as well. For example, in the case [Arp2/3] = 12.5 nM, when *L* = 9 µm dimerization is favorable for bundles comprising no more than *s* = 4 shells, and the highest energetic gain is obtained upon dimerization of bundles comprising of *s* = 2 shells. On the other hand, when *L* = 12 µm, the maximum number of shells in bundles capable of dimerization to is *s* = 5, and the optimal thickness is obtained for doubling *s* = 3 bundles. Fig. 6c also reveals the important role of [Arp2/3] on the length and thickness of the bundles. When [Arp2/3] = 100 nM a larger fraction of actin monomers (as compared to the case [Arp2/3] = 12.5 nM) are consumed during aster's assembly, leaving less monomers for bundle growth. This fact, as well as the initial *b* and *L*, are responsible for the shorter and thinner bundles corresponding to the high [Arp2/3] system, (see magnified box). In this case, the typical bundle consists of a small (s∼1) number of shells.

To derive the results shown in Fig. 6c we expressed the dimerization energy as a sum of bending and fascin linking energies: Δ*E_dim_* = *E_bend_*+Δ*E_fascin_*, both depending on *s*. To calculate the bending energy of a bundle upon joining another one we have used a simple geometrical model, whereby its bent portion, of length *l*, is represented as composed of two oppositely (and moderately) bent, but otherwise identical arcs, joining smoothly each other to an s-like shape (as may be seen in Fig. 6b). It can be shown that this implies *E_bend_* = *γ a* ^2^/*l* ^3^ (details will be published elsewhere). Here *a* is the distance between the origins of the bundles (*a* = *bs* for two neighboring bundles of size *s*), *γ* = 32*ξkT* where *ξ* is the persistence length of the bundle, *k* is Boltzmann's constant and *T* is the absolute temperature. Since fascin is abundant in the system, the actin filaments within bundles are tightly bound, and the bundles thus behave as semiflexible rods of thickness *s*, and persistence length *ξ_s_* = *π* ^2^ *s* ^4^ *ξ*_1_, where *ξ*_1_≈10 *µm* is the persistence length of a single actin filament.

The energy change upon linking two bundles is proportional to the length, *L-l*, of their straight and parallel portions (Fig. 6b), i.e., Δ*E_fascin_* = −(*L*−*l*)*ε*, where *ε* is the gain, per unit length, in the cohesive energy due to the fascin-F-actin bonds. Assuming that all possible actin-fascin contacts are saturated, and that actin filaments are hexagonally packed within a bundle, we find that for two cylindrical bundles which associate into a thicker cylindrical bundle *ε* = 0.2*πsε*_1_, where *ε*_1_ = 833*kT*/*µm* is the binding energy of fascin to a unit length of a single F-actin. Minimizing Δ*E_dim_* = *γb* ^2^ *s* ^2^/*l* ^3^−(*L*−*l*)*ε* with respect to *l*, we find the optimal length of the bent (‘root-like”, Fig. 6b) portion of the bundle: *l_s_* = *αb* ^1/2^ *s* ^1/2^, where *α* = (3*γ*/*ε*)^1/4^. Substituting *l_s_* into Δ*E*_dim_(*s*) we get Δ*E_dim_*(*s*) = *C_A_s* ^9/4^−*C_B_Ls* where 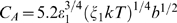 and *C_B_* = 0.2*πε*_1_.

We conclude this analysis by noting that from some point in time onwards, the bundles observed experimentally appear to continue elongating without changing their thickness. Based on our model we may conclude that this happens when the time scale of bundle bending fluctuations which lead to bundle-bundle association becomes long compared to the experimental time scale, or simply long relative to the time it takes to all actin monomers to join the growing bundles.

## Discussion

In this work we studied in detail the steady state structures formed in a simple *in vitro* system, in which Arp2/3 complex and fascin compete on their binding to actin filaments. Varying mixtures of actin, Arp2/3, and fascin were analyzed in order to elucidate their delicate interplay in determining which of the structures–asters, stars, or networks of bundles–appear in a given concentration regime.

We found that in the absence or at very low concentration of fascin the system is dominated by Arp2/3 complex nucleation and branching activities, resulting in the appearance of dense 3D aster-like networks of actin. Increasing fascin concentration induces phase changes, first to stars and then to network of bundles. A star is composed of a dense aster core with actin/fascin bundles emanating radially from its surface. Our experiments show that in the early stages of actin polymerization fascin is passive, while Arp2/3 mediates dense aster-like structures of actin, whose structure is very similar to the one observed in the absence of fascin. Fascin comes into play when actin filaments in the periphery of the aster get long enough, and can thus bend and associate with each other into bundles of parallel filaments, held together by fascin linkers. This is in accordance with *in vivo* experiments that emphasize the importance of filaments length in the creation of filopodia [16]. The aster-star transition appeared for all Arp2/3 concentrations that we have tested. The second transition, from stars to network of bundles, has only been observed at sufficiently low [Arp2/3]. In conclusion, we find that the system is more sensitive to phase changes when [Arp2/3] is low, and the concentration of fascin necessary to induce structural transformations is lower.

The competition between Arp2/3 and fascin is critical in determining the actin structures formed. This is because these two proteins are nucleators of different actin geometries; Arp2/3 complex promotes the formation of branched actin seeds [8] while fascin initiates unbranched bundle seeds [31]. The ability of fascin to nucleate actin bundles *in vitro*, was recently demonstrated in experiments showing that fascin enhances actin polymerization by promoting the formation of stable disc-like bundle nuclei. These nuclei are composed of short filaments (a few monomers long), which serve as seeds for subsequent actin polymerization [31]. In the presence of Arp2/3, this process competes with branch nucleation of actin. The rates of these two processes are expected to be regulated by the abundance of Arp2/3 complex and fascin.

In formulating our model for bundle formation and growth we assumed that only filaments originating at the surface of the aster take part in this process (similar to *in vivo* systems where filopodia originate in the vicinity of the plasma membrane). Filaments from the inner core of the aster cannot significantly contribute to this process, owing to severe excluded volume interactions on the bending of these highly branched, dense, and entangled filaments. We assumed that bundles begin to form when filaments in the aster surface are long enough so that their bending energy penalty is compensated by the gain of actin-fascin linking energy. Since the unbinding time of bundles is significantly longer than the polymerization time of filaments, their association is essentially irreversible, and thus cannot be adequately described based on thermodynamic equilibrium consideration. We have thus proposed a step-wise mechanism of irreversible assembly of single filaments/bundles into thicker bundles, followed by their elongation. The newly formed bundles elongate until their bending energy decreases, and their net association energy into a thicker bundle becomes energetically favorable, etc. This model can also explain the gradual thickening of newly formed bundles *in vivo*, forming Λ-precursors shaped roots [5]. In our experimental system, the elongation-thickening sequence terminates when all actin monomers are consumed. Based on this picture we could explain why at low [Arp2/3] the bundles emerging from the aster's core are both longer and thicker as compared to those observed in systems with a higher initial [Arp2/3].

The evolution of asters to stars is of direct biological relevance because of its similarity to lamellipodia to filopodia transition in cells. Recall that two structural parameters, *L* and *b*, characterize the periphery of aster's network. In our *in vitro* system, the length, *L*, of the (unbranched sections of the) surface filaments increases in time, while the spacing between their origins decreases. In living cells, analogous structural parameters may determine the conditions favoring the formation of filopodia. In the cellular system, filaments length (*L*) and the density of the lamellipodial network (characterized by *b*) is controlled by specific regulatory proteins, such as Arp2/3 complex, VASP and Dia2. In particular, Dia2 was recently shown to be recruited to lamellipodia, where it induces the formation of many long, unbranched filaments (thus locally increasing *L* and reducing *b*), which then gradually converged into filopodial bundles [24], [32]. This behavior is qualitatively consistent with our model, according to which an increasing *L* or decreasing *b*, favors the onset of bundle formation. By recruiting proteins like Dia2 to specific sites along lamellipodia, such a mechanism may enable the cell to control the localization of filopodia along its leading edge.

To conclude, using a minimal model system containing only Arp2/3 complex, actin and fascin we were able to mimic complex events which take place at the leading edge of cells. Compared to a cell this system is simple and easily controlled, making it possible to characterize the roles of a limited set of proteins in the higher order assembly of actin filaments. In the future, it will be interesting to measure the size of the actin/fascin bundle using high resolution electron microscopy, and correlate it with the properties of the aster network structure (e.g., density of the actin network, filament length and orientation at the aster periphery). It is worthwhile to test the effects of mDia2 and capping proteins concentrations on bundle formation and dimensions. Finally, quantitative information from *in vivo* studies will be of great value, especially regarding the correlation between filopodia thicknesses and structural properties of the lamellipodial network.

## Materials and Methods

### Protein purification

Actin was purified from rabbit skeletal muscle acetone powder [33]. Recombinant WASp-VCA [26] and fascin (recombinant fascin was prepared by a modification of the method of [34]) were expressed in *E. coli* as GST fusion proteins. Arp2/3 complex was purchased from Cytoskeleton Co. Actin was labeled on Cys374 with Alexa fluor 568 or 488 (Invitrogen, Co.).

### Motility assay

The motility medium contained 10 mM HEPES, pH = 7.7, 1.7 mM Mg-ATP, 5.5 mM DTT, 0.12 mM Dabco [an anti-bleaching reagent], 0.1 M KCl, 1 mM MgCl_2_, 1% BSA and various concentrations of G-actin, Arp2/3 complex, VCA, and fascin.

### Microscopy

Actin assembly was monitored for about an hour by fluorescence with an Olympus IX-71 microscope. The labeled actin fraction was 1/40 and the temperature was 22°C. Time-lapse images were acquired using an Andor DV887 EMCCD camera (Andor Co., England). Data acquisition and analysis was performed using METAMORPH (Universal Imaging Co.).

## Supporting Information

Appendix S1 (0.38 MB DOC)

Figure S1 Conditions are: 7 µM G-actin, (a–b) 12.5 nM Arp2/3 complex, and 25 nM GST-VCA and (c–d) 100 nM Arp2/3, and 200 nM GST-VCA. The amount of fascin required inducing transition from aster (a and c) to stars (b and d) ranges between 5 to 6 nM and between 6.5 to 7 nM at 12.5 nM and 100 nM Arp2/3 complex, respectively. The insets in b and d show zoom-in images of the bundles emanating from the stars at each [Arp2/3]. At the transition, the bundles emanating from the stars at lower [Arp2/3] (b) are thicker than those originating from the aster core at 100 nM Arp2/3 (d). Bars are 10 µm.
(2.78 MB TIF)
