# Supplementary material for: Arp2/3 Branched Actin Network Mediates Filopodia-Like Bundles Formation In Vitro
Source: PLoS One. 2008 Sep 29;3(9):e3297. doi: 10.1371/journal.pone.0003297 (PMC2538570; doi:10.1371/journal.pone.0003297)
Supplement: Appendix S1 — (0.38 MB DOC) [file pone.0003297.s001.doc]

**Supporting Information**

**Appendix 1** In the KMC simulations, asters evolve from an actin dimer nucleus. The kinetic processes considered are polymerization at barbed ends and branching on existing filaments. Excluded volume interactions are taken into account so that filaments can not intersect each other. To account for the experimental fact that many asters grow simultaneously in solution, one aster is grown inside a simulation box, whose volume is chosen as the average volume available, experimentally, per aster. The concentrations of free actin monomers and Arp2/3 complexes decrease over time owing to their consumption in the course of polymerization and branching. In our simulations it is assumed that the rate of diffusion of these particles is larger than the rates of polymerization and branching, thus ensuring that their momentary spatial distributions are always uniform. It may be noted, however, that under certain conditions the spatial dependence G-actin concentration can not be ignored [S1]. Similar to the experimental conditions, the initial concentrations in the simulations are [G-actin] = [A] = 7.5µM, and [Arp2/3]=12.5nM and 100nM. For more details on the simulations see Haviv et. al [S2].

As noted earlier, the values of *b* and *L*, which enter our model, were determined based on the simulations. To this end we have defined an outer aster layer of thickness *L*, from which we collect the data (Fig. 6a). We choose a layer width that equals the average length of filaments that originate on the aster surface and have a single daughter branch. It should be noted, that other choices proportional to the branch spacing were examined and the qualitative results do not depend on this choice. For this layer we have also determined the average distance, *b*, between the pointed ends of the peripheral filaments.

**Figure S1** Conditions are: 7μM G-actin, (a-b) 12.5nM Arp2/3 complex, and 25nM GST-VCA and (c-d) 100nM Arp2/3, and 200nM GST-VCA. The amount of fascin required inducing transition from aster (a and c) to stars (b and d) ranges between 5 to 6nM and between 6.5 to 7nM at 12.5nM and 100nM Arp2/3 complex, respectively. The insets in b and d show zoom-in images of the bundles emanating from the stars at each [Arp2/3]. At the transition, the bundles emanating from the stars at lower [Arp2/3] (b) are thicker than those originating from the aster core at 100nM Arp2/3 (d). Bars are 10μm.

**Supplementary references**

**S1.** [Dickinson RB](http://www.ncbi.nlm.nih.gov/sites/entrez?Db=pubmed&Cmd=Search&Term="Dickinson RB"%5BAuthor%5D&itool=EntrezSystem2.PEntrez.Pubmed.Pubmed_ResultsPanel.Pubmed_DiscoveryPanel.Pubmed_RVAbstractPlus), [Purich DL](http://www.ncbi.nlm.nih.gov/sites/entrez?Db=pubmed&Cmd=Search&Term="Purich DL"%5BAuthor%5D&itool=EntrezSystem2.PEntrez.Pubmed.Pubmed_ResultsPanel.Pubmed_DiscoveryPanel.Pubmed_RVAbstractPlus) (2006) Diffusion rate limitations in actin-based propulsion of hard and deformable particles. [Biophys J.](javascript:AL_get(this, 'jour', 'Biophys J.');) 91(4): 1548-1563.

**S2.** Haviv L, Brill-Karniely Y, Mahaffy R, Backouche F, Ben-Shaul A, et al. (2006) Reconstitution of the transition from lamellipodium to filopodium in a membrane-free system. Proc Natl Acad Sci U S A 103(13): 4906-4911. 10.1073/pnas.0508269103**.**

**
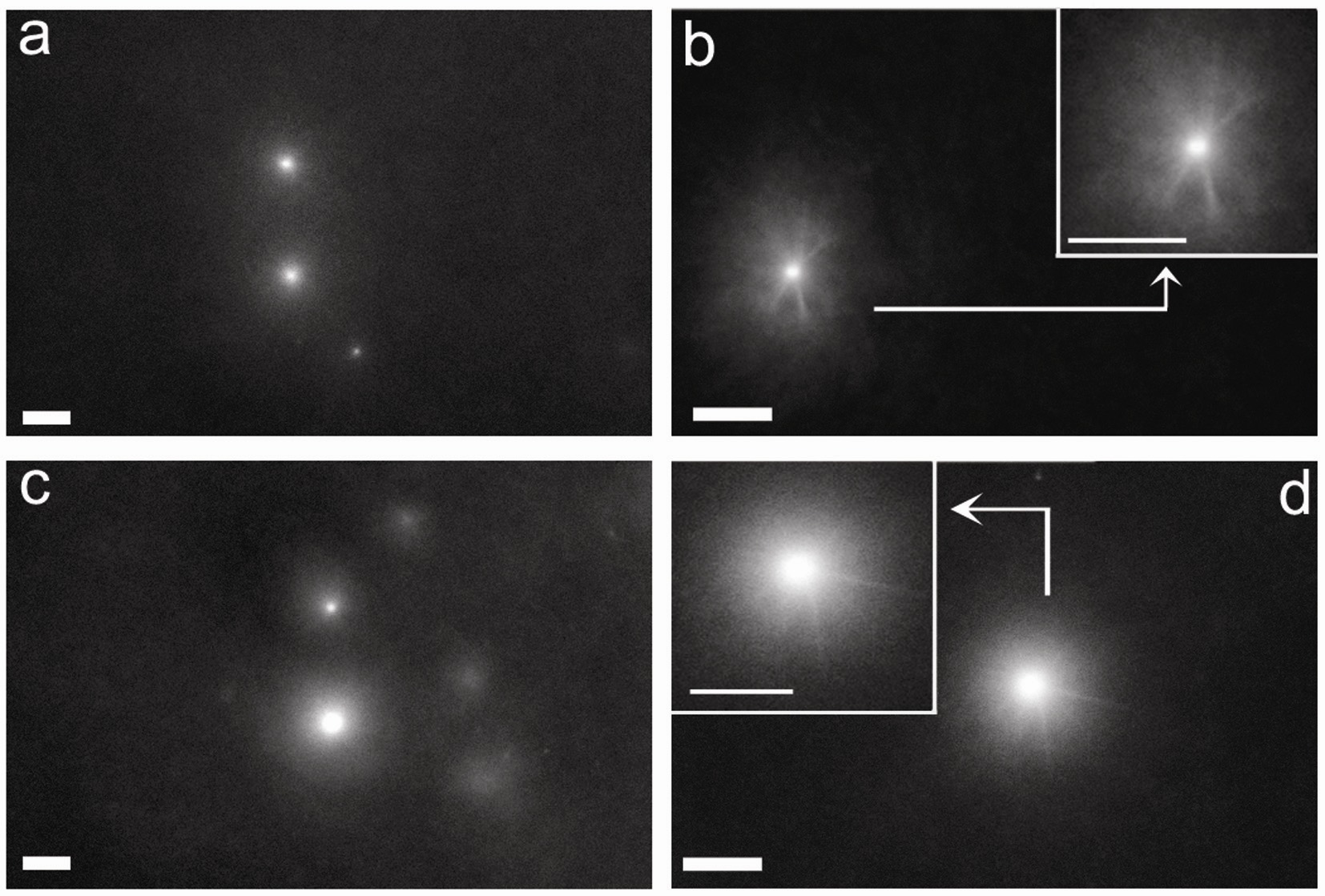
**

Ideses et al. Figure. S1
