# Supplementary figures and images for: Arp2/3 Branched Actin Network Mediates Filopodia-Like Bundles Formation In Vitro
Source: PLoS One. 2008 Sep 29;3(9):e3297. doi: 10.1371/journal.pone.0003297 (PMC2538570; doi:10.1371/journal.pone.0003297)

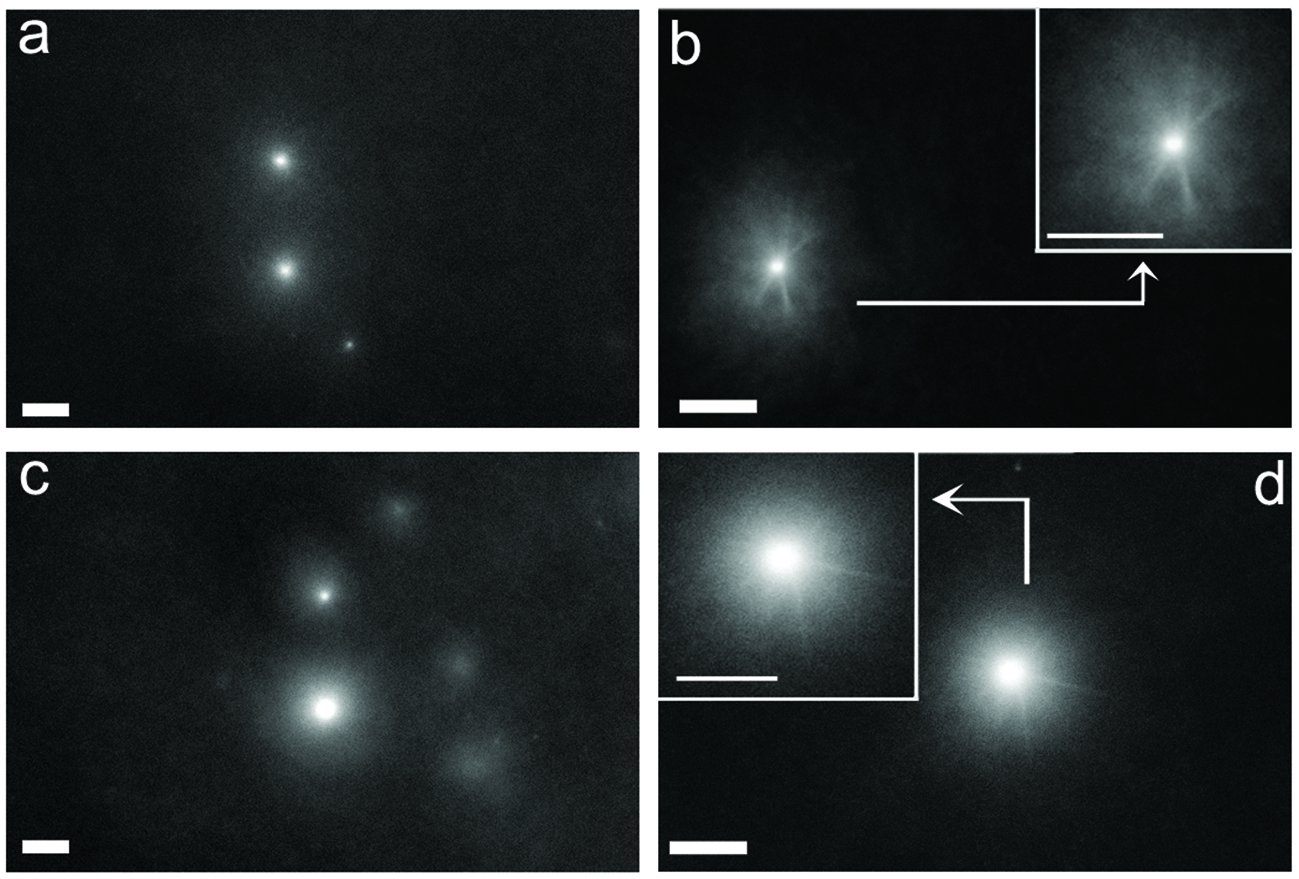

Supplement: Figure S1 — Conditions are: 7 µM G-actin, (a–b) 12.5 nM Arp2/3 complex, and 25 nM GST-VCA and (c–d) 100 nM Arp2/3, and 200 nM GST-VCA. The amount of fascin required inducing transition from aster (a and c) to stars (b and d) ranges between 5 to 6 nM and between 6.5 to 7 nM at 12.5 nM and 100 nM Arp2/3 complex, respectively. The insets in b and d show zoom-in images of the bundles emanating from the stars at each [Arp2/3]. At the transition, the bundles emanating from the stars at lower [Arp2/3] (b) are thicker than those originating from the aster core at 100 nM Arp2/3 (d). Bars are 10 µm. (2.78 MB TIF) [file pone.0003297.s002.tif]
